# Role of peripheral markers of inflammation in cognitive dysfunction in schizophrenic patients: a systematic review

**DOI:** 10.1192/j.eurpsy.2023.2241

**Published:** 2023-07-19

**Authors:** F. Ben Othman, A. Aissa, A. Adouni, Z. Yosra, U. Ouali, R. Jomli

**Affiliations:** Psychiatry, Razi Hospital, Tunis, Tunisia

## Abstract

**Introduction:**

Schizophrenia (SCZ) is a chronic mental illness characterized by a rich and diverse symptomatology. A generalized cognitive deficit has been widely recognized among the symptoms of this disease. Several authors have studied the relationship between peripheral markers of inflammation and cognitive dysfunctions in order to explain the etiopathogeny of these disorders.

**Objectives:**

The aim of our study is to better comprehend the nature of the relation between peripheral markers of inflammation and cognitive dysfunctions.

**Methods:**

A systematic review of the literature was conducted following the guidelines provided by the PRISMA method. We performed a systematic search focused on two automated bibliographic databases: Pubmed and Google Scholar including the following keywords: “inflammation”, “schizophrenia”, “cognition”

**Results:**

A total of 17 articles were included.

Significant relations with cognitive function were reported with IL-6, IL-18, IL-2, IL-8, tumor necrosis factor α (TNF-α ) and chemokines. Memory was the cognitive domain where the most significant relations with cytokines were objectified.

BDNF levels were correlated with cognitive tests in 5 studies of SCZ populations. The domains concerned were inhibition, flexibility, verbal fluency, verbal memory, attention, and processing speed.

Elevated CRP in patients with SCZ was reported by all studies and a significant relation with cognition in 3 studies. This relations is objectified in the areas of memory, executive functions and processing speed.

The relations between CRP, BDNF, cytokines and cognitive functions was inconsistent across studies.

**Conclusions:**

The majority of the results observed during the review were in favor of a significant relation between CRP, BDNF and cytokines. Nevertheless, these results were not constant and heterogeneous. It would be interesting to better explore the nature of this relation through prospective studies in order to establish therapeutic perspectives.

**Disclosure of Interest:**

None Declared

